# Tregs epigenetically reprogrammed from autoreactive effector T cells mitigate established autoimmunity

**DOI:** 10.1172/jci.insight.185581

**Published:** 2025-07-31

**Authors:** Tyler R. Colson, James J. Cameron, Hayley I. Muendlein, Mei-An Nolan, Jamie L. Leiriao, James H. Kim, Alexander N. Poltorak, Xudong Li

**Affiliations:** 1 Graduate Program in Immunology, Tufts Graduate School of Biomedical Sciences, Boston, Massachusetts, USA.; 2 Department of Immunology, Tufts University School of Medicine (TUSM), Boston, Massachusetts, USA.

**Keywords:** Autoimmunity, Immunology, Inflammation, Autoimmune diseases, Epigenetics, T cells

## Abstract

Reprogramming autoreactive CD4^+^ effector T (Teff) cells into immunosuppressive Tregs represents a promising strategy for treating established autoimmune diseases. However, the stability and function of such reprogrammed Tregs under inflammatory conditions remain unclear. Here, we show that demethylation of core Treg identity genes in Teff cells yields lineage-stable effector T cell reprogrammed Tregs (ER-Tregs). A single adoptive transfer of ER-Tregs not only prevents autoimmune neuroinflammation in mice when given before disease onset but also arrests its progression when administered after onset. Compared with Foxp3-overexpressing Teff cells, induced Tregs from naive precursors, and endogenous Tregs, ER-Tregs provide superior protection against autoimmune neuroinflammation. This enhanced efficacy stems from their inherited autoantigen specificity and selectively preserved effector cell transcriptional programs, which together bolster their fitness in inflammatory environments and enhance their suppressive capacity. Our results establish epigenetic reprogramming of autoreactive Teff cells as an effective approach to generate potent, stable Tregs for the treatment of refractory autoimmune conditions.

## Abstract 2

Autoreactive effector T cells can be epigenetically reprogrammed into lineage-stable regulatory T cells to effectively control established autoimmune inflammation

## Graphical abstract

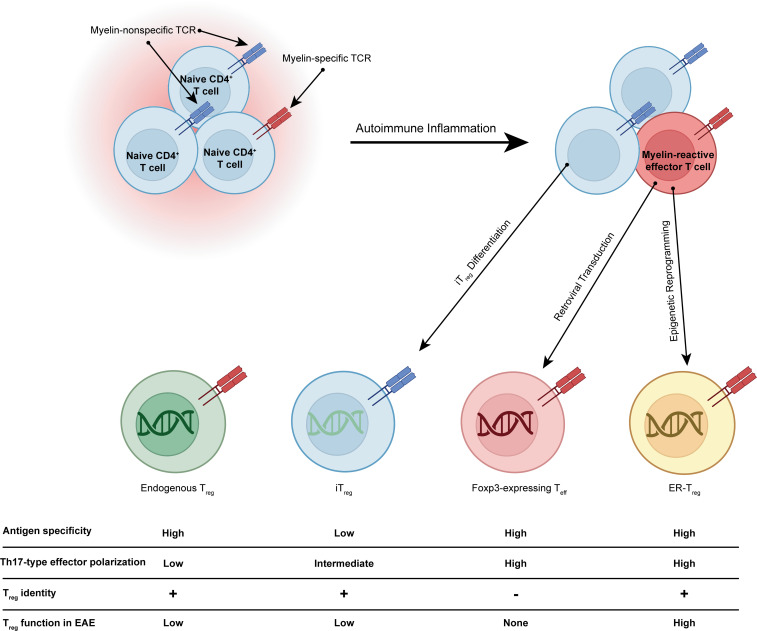

## Introduction

CD4^+^ regulatory T cells (Tregs) expressing the transcription factor Foxp3 play an essential role in immune homeostasis by preventing autoimmunity against self-antigens and curtailing deleterious immune responses toward environmental antigens (1, 2). However, naturally occurring endogenous Tregs (nTregs) are often inadequate at suppressing ongoing inflammation in established autoimmune diseases (3, 4), limiting their therapeutic efficacy (5). This highlights the urgent need to better understand the mechanisms underlying disease-associated Treg functional deficiencies so that novel approaches can be developed to reinvigorate their function for the treatment of autoimmune diseases.

Treg functional deficiency can arise from either inadequate Treg suppressor function on a per-cell basis or a paucity of autoantigen specific Tregs. The latter may result from impaired differentiation and lineage stability of autoantigen specific Tregs, leading to their diversion into effector CD4^+^ T (Teff) cell fates (6–11). In this regard, reprogramming autoreactive CD4^+^ Teff cells into Tregs for adoptive cell therapy presents an attractive option for restoring Treg function. This approach could generate Tregs that share the autoantigen specificities of their target Teff cells, potentially enhancing antigen specific suppression (12). However, the development of this approach is hindered by the lack of an effective method to convert CD4^+^ Teff cells into bona fide Tregs that exhibit Treg-specific gene expression and function (12–14).

The maintenance of Treg cellular identity and suppressive function depends on the stable expression of the transcription factor Foxp3, along with Foxp3-independent core identity genes (15–19). However, whether stable induction of Treg identity genes in committed CD4^+^ Teff cells can establish bona fide Treg identity and suppressive capacity remains unresolved. Preexisting Teff gene expression may antagonize Treg identity establishment (20), raising concerns that residual effector signatures could destabilize lineage commitment or impair suppressor activity in reprogrammed Treg populations. Alternatively, the inherent Teff gene expression — when combined with the reprogramming process — could actually enhance suppressor function by imparting an effector Treg-like gene expression profile, where heightened expression of specific Teff genes correlates with superior suppressive capabilities (21–34). Clarifying these possibilities is crucial not only for advancing this therapeutic strategy but also for providing novel insights into how the core Treg identity gene program interacts with effector gene expression to shape Treg function. Nevertheless, the fundamental question of whether the core Treg identity genes can be stably activated in CD4^+^ Teff cells remains unresolved, hindering deeper exploration of this approach.

Treg gene expression is controlled by intricate epigenetic mechanisms, including DNA methylation and demethylation (11, 35–39). During Treg development, the stable activation of *Foxp3* and other core Treg identity genes requires the demethylation of DNA at cytosine-guanine (CpG) motifs in conserved *cis*-regulatory regions (16, 40–42), presenting therapeutic targets for boosting Treg function. Indeed, pharmacologically enhancing DNA demethylation in preexisting Tregs augments their fitness and function and accelerates repair of experimental lung injury (43). Importantly, we and others have shown that demethylation at conserved noncoding sequence 2 (CNS2) within the *Foxp3* gene stabilizes its expression in effector Tregs by counteracting the transcriptional inhibitory effects of TCR and proinflammatory cytokine signaling (44, 45). These *Foxp3*-inhibitory signals are also highly active in Teff cells (46–48), suggesting that induction of stable *Foxp3* expression in CD4^+^ Teff cells likely requires CNS2 demethylation. Furthermore, we recently demonstrated that CNS2 demethylation requires sustained *Foxp3* transcriptional activation (49), indicating a positive feedback loop where *Foxp3* transcriptional activation and DNA demethylation mutually reinforce each other, facilitating Treg lineage commitment during differentiation. These findings suggest that fostering sustained transcriptional activation of *Foxp3* and other core Treg identity genes in an environment conducive to DNA demethylation may enable epigenetic reprogramming of CD4^+^ Teff cells into Tregs.

To determine whether autoreactive CD4^+^ Teff cells can be reprogrammed into bona fide Tregs for mitigating established autoimmunity, we developed an approach to achieve stable demethylation of Treg identity genes in CD4^+^ Teff cells. Our findings show that this approach generates bona fide Tregs, which we term effector T cell reprogrammed Tregs (ER-Tregs). These ER-Tregs exhibit a superior ability to ameliorate established autoimmune neuroinflammation compared with CD4^+^ Teff cells forced to express Foxp3 exogenously, induced Tregs (iTregs) derived from naive CD4^+^ T (Tn) cells, and endogenous nTregs. The autoantigen specificity inherited by ER-Tregs from autoreactive CD4^+^ Teff cells enables antigen-specific suppression of autoimmune inflammation without compromising normal immune function. Additionally, the selective inheritance of parental Teff gene expression confers ER-Tregs superior fitness and suppressor functionality under inflammatory conditions. Thus, demethylation of Treg identity genes in autoreactive CD4^+^ Teff cells can establish a bona fide Treg gene expression program, giving rise to Tregs capable of quelling established autoimmune inflammation.

## Results

### Epigenetic reprogramming enables stable induction of Foxp3 and other core Treg identity genes.

**Figure 1:**
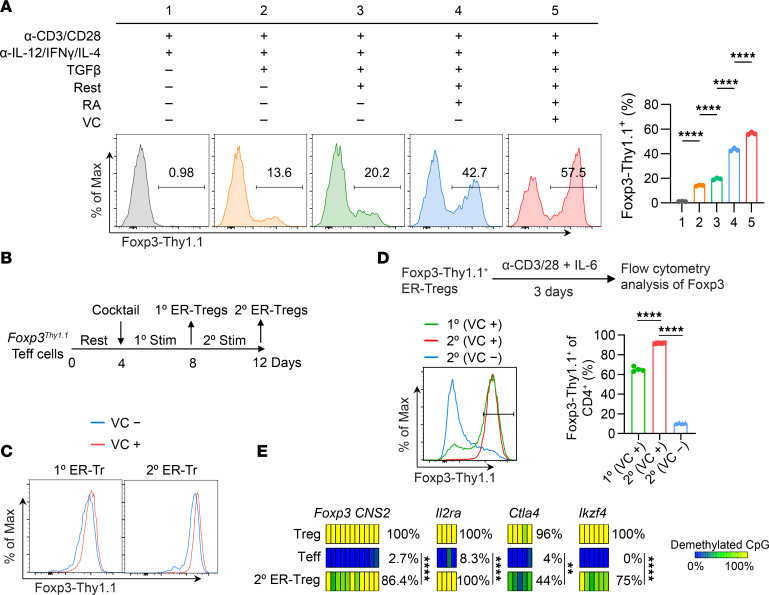
Epigenetic reprogramming of CD4^+^ Teff cells into Tregs. (**A**) Flow cytometry of Foxp3-Thy1.1 induction in CD4^+^ Teff cells isolated from *Foxp3^Thy1.1^* mice on day 7 following immunization with MOG/CFA and subsequently activated with anti-CD3/CD28 microbeads for 4 days under indicated conditions. Rest indicates resting Teff cells for 4 days prior to their activation. RA indicates retinoic acid. VC indicates vitamin C. (**B**) Schematic of epigenetic reprogramming of CD4^+^ Teff cells into ER-Tregs. (**C**) Flow cytometry of Foxp3-Thy1.1 expression in 1° and 2° ER-Tregs generated in the presence or absence of VC. (**D**) Flow cytometry of Foxp3-Thy1.1 expression in 1° and 2° ER-Tregs generated in the presence or absence of VC and subsequently restimulated for 3 days in the presence of IL-6. (**E**) Heatmaps of CpG demethylation patterns at specific loci in indicated cell types, analyzed with bisulfite-sequencing. Each bar represents a CpG site. Data are shown as mean ± SEM. **P < 0.01, ****P < 0.0001, 1-way ANOVA and Holm-Šídák test in **A** and **D**, and 2-way ANOVA in **E**.

*Foxp3* expression is essential for establishing Treg cellular identity (17–19). To induce this identity in proinflammatory CD4^+^ Teff cells from mice with experimental autoimmune encephalomyelitis (EAE), we first optimized conditions for efficient *Foxp3* induction. We purified CD4^+^Foxp3-Thy1.1^−^CD44^hi^ Teff cells from *Foxp3^Thy1.1^* reporter mice (50) immunized with myelin oligodendrocyte glycoprotein (MOG) peptide emulsified in complete Freund’s adjuvant (CFA). When activated in vitro under an iTreg differentiation condition consisting of IL-2, TGF-β, and neutralizing antibodies against proinflammatory cytokines IL-12, IFN-γ, and IL-4 (51, 52), approximately 14% of the Teff cells began expressing Thy1.1 (Figure 1A). Preresting Teff cells before activation — along with the addition of retinoic acid (RA) to promote *Foxp3* induction (53–56) and vitamin C (VC) to facilitate DNA demethylation (38, 39) — progressively enhanced *Foxp3* induction, resulting in nearly 60% of cells expressing Foxp3 (Figure 1A).

Sustained transcriptional activation of *Foxp3* enhances its epigenetic stabilization by promoting CNS2 demethylation (49). To test whether maintaining *Foxp3* activation in ER-Tregs through restimulation with the ER-Treg reprogramming cocktail improves *Foxp3* stability, we restimulated the cells in this context (Figure 1B). Both restimulation and the inclusion of VC during reprogramming increased *Foxp3* expression (Figure 1C) and, more importantly, improved its stability when ER-Tregs were subsequently exposed to the proinflammatory cytokine IL-6 (Figure 1D). Notably, restimulated ER-Tregs displayed significant DNA demethylation at Treg-specific demethylation regions (TSDRs), including *Foxp3 CNS2*, *Il2ra*, *Ctla4*, and *Ikzf4* (16), compared with parental Teff cells (Figure 1E). Collectively, these findings suggest that both features defining Treg cellular identity — stable *Foxp3* expression and epigenetic activation of core Treg identity genes — can be successfully established in committed CD4^+^ Teff cells by epigenetic reprogramming.

### Adoptive transfer of ER-Tregs prevents EAE development and ameliorates established EAE.

**Figure 2:**
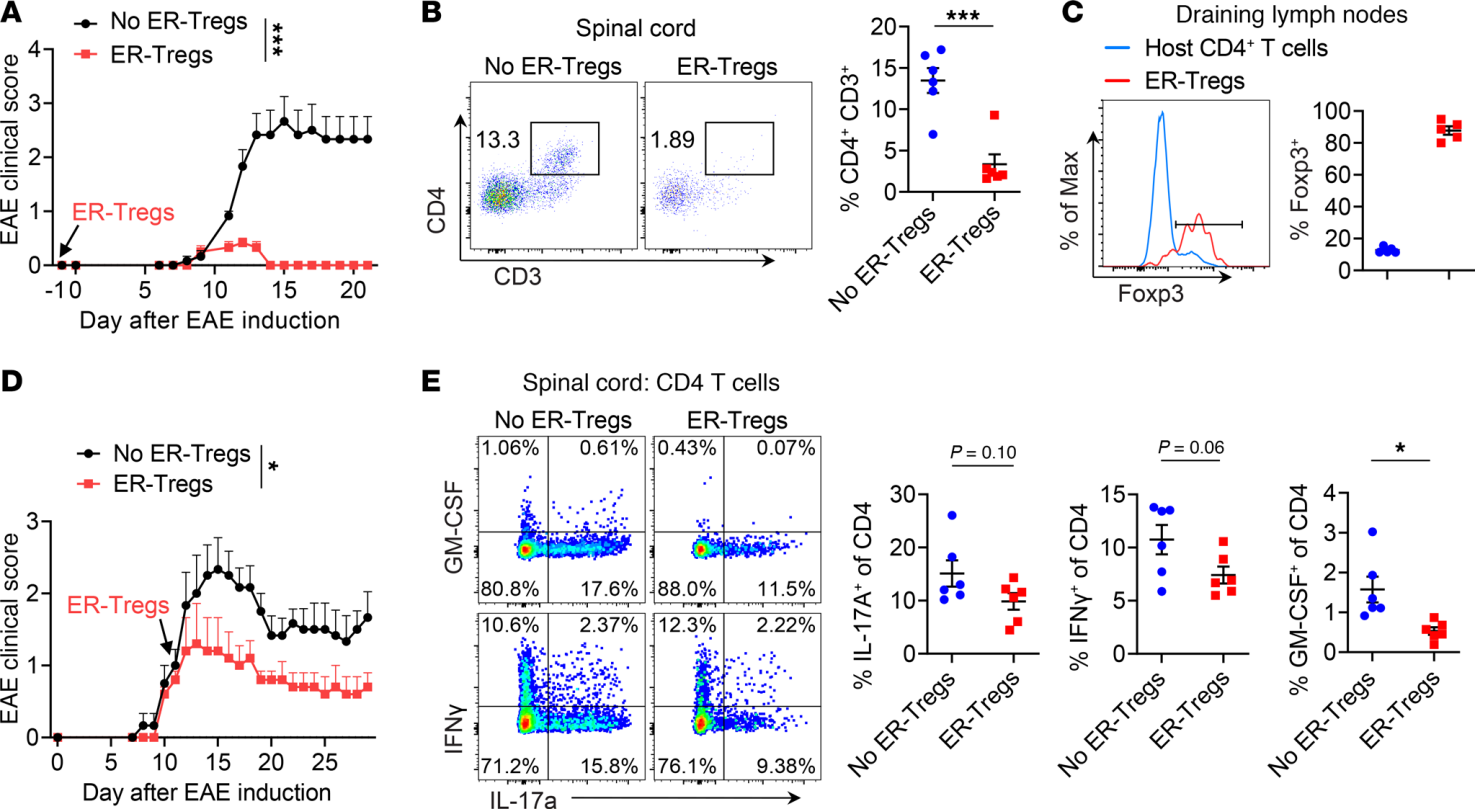
Adoptive transfer of ER-Tregs prevents EAE development and ameliorates established EAE. (**A**–**C**) EAE was induced via MOG/CFA immunization in CD45.2^+^ mice with or without adoptive transfer of ER-Tregs reprogrammed from MOG/CFA-primed CD45.1^+^*Foxp3^Thy1.1^* CD4^+^ Teff cells, administered 1 day prior to immunization. Flow cytometry analyses were conducted at 21 days postimmunization (dpi). *n* = 6 per group. Data are representative of 2 independent experiments. (**A**) EAE disease curve. (**B**) Flow cytometry analysis of the frequencies of spinal cord CD4^+^ T cells. (**C**) Flow cytometry of Foxp3 expression in host CD4^+^ T cells and transferred ER-Tregs within the draining lymph nodes (LNs) of mice that received ER-Tregs. (**D** and **E**) EAE was induced via MOG/CFA immunization in CD45.2^+^ mice with or without adoptive transfer of ER-Tregs reprogrammed from MOG/CFA-primed CD45.1^+^*Foxp3^Thy1.1^* Teff cells, administered at 11 dpi. Flow cytometry analyses were conducted at 29 dpi. *n* = 6 per group. Data are representative of 2 independent experiments. (**D**) EAE disease curve. (**E**) Flow cytometry of IFN-γ and GM-CSF expression in CD4^+^ T cells in spinal cord. Data are shown as mean ± SEM. *P < 0.05, ***P < 0.001, unpaired 2-tailed *t* test of AUC in (**A** and **D**) and unpaired 2-tailed *t* test in (**B** and **E**).

To assess the therapeutic potential of ER-Tregs in curbing autoimmune inflammation, we adoptively transferred ER-Tregs derived from MOG/CFA-primed CD4^+^ Teff cells into CD45 congenically distinct mice 1 day before inducing EAE via MOG/CFA immunization and pertussis toxin (PT; List Labs) injections. Remarkably, ER-Treg transfer nearly abolished clinical manifestations of EAE, in stark contrast to the rapid disease progression observed in control animals (Figure 2A). Consistent with this protection, CD4^+^ T cell infiltration into the spinal cord was significantly reduced in ER-Treg–treated mice (Figure 2B). Approximately 90% of transferred ER-Tregs maintained Foxp3 expression, demonstrating robust in vivo stability under inflammatory conditions (Figure 2C). Although ER-Tregs comprised only about 2% of all Tregs in the draining lymph nodes (Supplemental Figure 1A; supplemental material available online with this article; https://doi.org/10.1172/jci.insight.185581DS1), they exhibited a significantly higher frequency of RORγt^+^ populations and a trending increase in RORγt^+^c-MAF^+^ populations compared with endogenous Tregs (Supplemental Figure 1B). This distinct phenotypic profile, combined with their scarcity, collectively suggests that ER-Tregs possess enhanced per-cell suppressive potency compared with endogenous Tregs under inflammatory conditions.

To assess the suppressive capacity of ER-Tregs in established EAE, we adoptively transferred ER-Tregs derived from MOG/CFA-primed CD4^+^ Teff cells into CD45 congenically distinct mice at disease onset (clinical score ~1). ER-Treg transfer attenuated disease progression (Figure 2D) and significantly reduced spinal cord infiltration by GM-CSF–producing CD4^+^ Teff cells (Figure 2E), a key driver of neuroinflammation (57–59). Detectable ER-Tregs were observed in only 2 recipients, likely reflecting the contraction of the transferred population as inflammation resolved. In these mice, ER-Tregs accounted for approximately 20% of spinal cord Foxp3^+^ cells (Supplemental Figure 1C). Strikingly, spinal cord ER-Tregs exhibited a higher frequency of RORγt^+^ and RORγt^+^c-MAF^+^ subsets compared with endogenous Tregs (Supplemental Figure 1D), further underscoring their distinct phenotype. Notably, ER-Tregs lacked expression of inflammatory cytokines IFN-γ or GM-CSF, although about 20% produced IL-17A (Supplemental Figure 1E). Collectively, these findings demonstrate that ER-Tregs retain robust lineage stability and suppressive potency in vivo, even within an established inflammatory niche, with a single transfer sufficient to ameliorate ongoing autoimmune pathology.

### Foxp3 expression is required but not sufficient for ER-Treg suppressor function.

**Figure 3:**
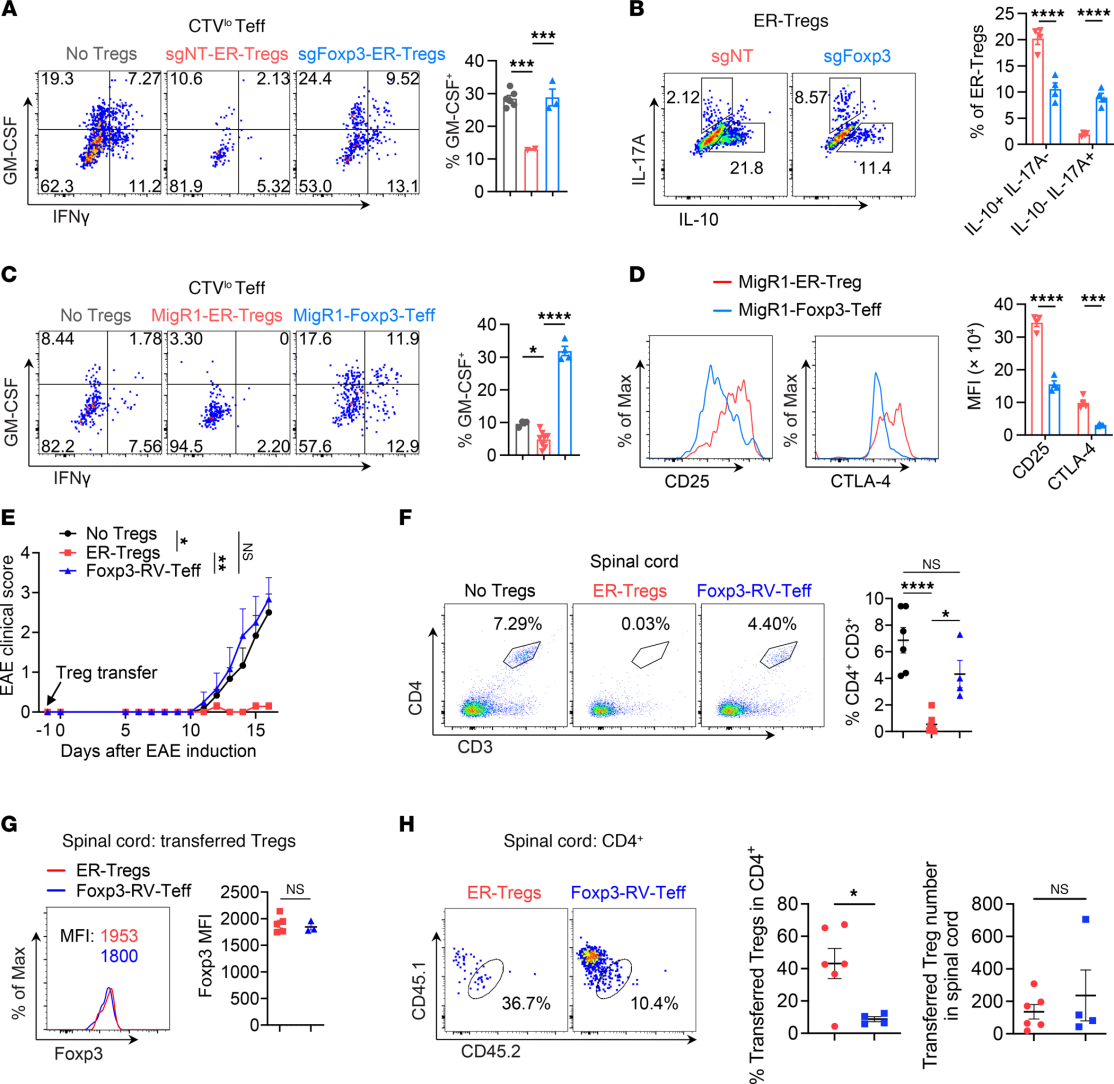
Foxp3 expression is required but not sufficient for the suppressor function of ER-Tregs. (**A**) Flow cytometry of IFN-γ and GM-CSF expression in CTV^lo^CD4^+^ Teff cells, cocultured for 3 days with T cell–depleted splenocytes serving as antigen presenting cells (APCs), in the presence of MOG and the presence or absence of *Foxp3^Thy1.1^ R26^Cas9^* ER-Tregs transduced with the retroviral vector (RV) expressing sgRNA targeting Foxp3 (sgFoxp3) or the nontargeting sgRNA (sgNT). (**B**) Flow cytometry of IL-10 and IL-17A expression in ER-Tregs as in **A**. (**C**) Flow cytometry of IFN-γ and GM-CSF expression in CTV^lo^CD4^+^ Teff cells, cocultured for 3 days with APCs, in the presence of MOG and the presence or absence of Foxp3^Thy1.1^ ER-Tregs transduced with MigR1 empty vector or CD4^+^ Teff cells forced to express Foxp3 via retroviral transduction with MigR1-Foxp3. (**D**) Flow cytometry of CD25 and CTLA-4 expression in MigR1-ER-Tregs and MigR1-Foxp3 CD4^+^ Teff cells as in **C**. (**E**–**G**) EAE was induced via MOG/CFA immunization in CD45.2^+^ mice with or without adoptive transfer of CD45.1^+^*Foxp3^Thy1.1^* MOG/CFA-primed CD4^+^ Teff cells reprogrammed into ER-Tregs or forced to express Foxp3 via retroviral transduction (Foxp3-RV-Teff), administered 1 day prior to immunization. Flow cytometry analyses were conducted at 16 dpi. *n* = 6 per group. (**E**) EAE disease curve. (**F**) Flow cytometry analysis of the frequencies of spinal cord CD4^+^ T cells. (**G**) Flow cytometry of Foxp3 expression in adoptively transferred ER-Tregs and Foxp3-RV CD4^+^ Teff cells in the spinal cord. (**H**) Flow cytometry of the frequencies and numbers of ER-Tregs or FRV-Tregs in the spinal cord. Data are shown as mean ± SEM. *P < 0.05, **P < 0.01, ***P < 0.001, ****P < 0.0001, 1-way ANOVA and Holm-Šídák test in (**A**, **C**, **E**, and **F**) and unpaired 2-tailed *t* test in (**B**, **D**, **G,** and **H**).

While Foxp3 expression is essential for the development and function of most endogenous Tregs (17–19), a recent study demonstrated that Foxp3 is dispensable for the fitness of microbiota-dependent peripherally induced Tregs (pTregs) and their ability to suppress colonic T cell expansion (60). To determine whether *Foxp3* activation is necessary for ER-Treg suppressor function, we used CRISPR/Cas9 to ablate *Foxp3* in *Foxp3^Thy1.1^R26^Cas9^* ER-Tregs. We transduced these cells with a retroviral vector expressing a single guide RNA targeting *Foxp3* (sgFoxp3) and compared their capacity to suppress GM-CSF expression in MOG/CFA-primed responder CD4^+^ Teff cells to that of ER-Tregs transduced with a nontargeting sgRNA (sgNT). *Foxp3* ablation completely abolished ER-Treg–mediated suppression of GM-CSF in CD4^+^ Teff cells following MOG stimulation (Figure 3A). Moreover, Foxp3-deficient ER-Tregs exhibited increased IL-17A expression (Figure 3B), consistent with studies showing that Foxp3 is critical for repressing IL-17A in pTregs (60). In addition, *Foxp3* ablation led to reduced IL-10 expression in ER-Tregs (Figure 3B). Collectively, these results indicate that activation of *Foxp3* is indispensable for the suppressive function of ER-Tregs.

To investigate whether epigenetic activation of Foxp3-independent Treg identity genes (Figure 1E) is essential for ER-Treg suppressive function, we forced Foxp3 expression in CD4^+^ Teff cells via retroviral transduction and assessed their regulatory capacity. In contrast to ER-Tregs, Foxp3-expressing Teff cells failed to suppress GM-CSF production in responder CD4^+^ Teff cells upon MOG stimulation. Instead, they amplified inflammatory responses, likely due to their inherently elevated expression of proinflammatory cytokines (Figure 3C and Supplemental Figure 2A). Furthermore, these cells exhibited markedly reduced expression of critical Treg effector molecules, including CD25 and CTLA-4, which are encoded by TSDR-containing *Il2ra* and *Ctla4*, respectively (61, 62) (Figure 3D).

To evaluate the in vivo relevance of these findings, we compared the therapeutic efficacy of Foxp3-expressing Teff cells with that of ER-Tregs in EAE. Adoptive transfer of Foxp3-expressing Teff cells neither attenuated disease progression nor reduced CD4^+^ T cell infiltration in the spinal cord (Figure 3, E and F). Although these cells expressed Foxp3 at levels comparable with ER-Tregs (Figure 3G), their relative abundance in the spinal cord was significantly lower (Figure 3H). These results underscore the necessity of Foxp3-independent epigenetic reprogramming for effective ER-Treg functionality.

To evaluate differences between Foxp3-expressing Teff cells and ER-Tregs, we directly compared their fitness and phenotype in immunocompetent EAE hosts. Foxp3-expressing Teff cells displayed reduced splenic engraftment, lower proliferation (as indicated by Ki-67 staining), and diminished expression of Helios and CD25 compared with ER-Tregs (Supplemental Figure 2, B–F). Given that Helios (encoded by *Ikzf2*) promotes Treg stability and survival (63, 64) and CD25 (encoded by *Il2ra*) enhances IL-2–dependent survival and function (62, 65, 66), the diminished suppressive capacity of Foxp3-expressing Teff cells, thus, likely results from inadequate epigenetic priming of critical genes (e.g., *Helios*, *Il2ra*), thereby compromising their resilience in inflammatory environments.

### Inherited autoantigen specificity confers superior functionality to ER-Tregs.

**Figure 4:**
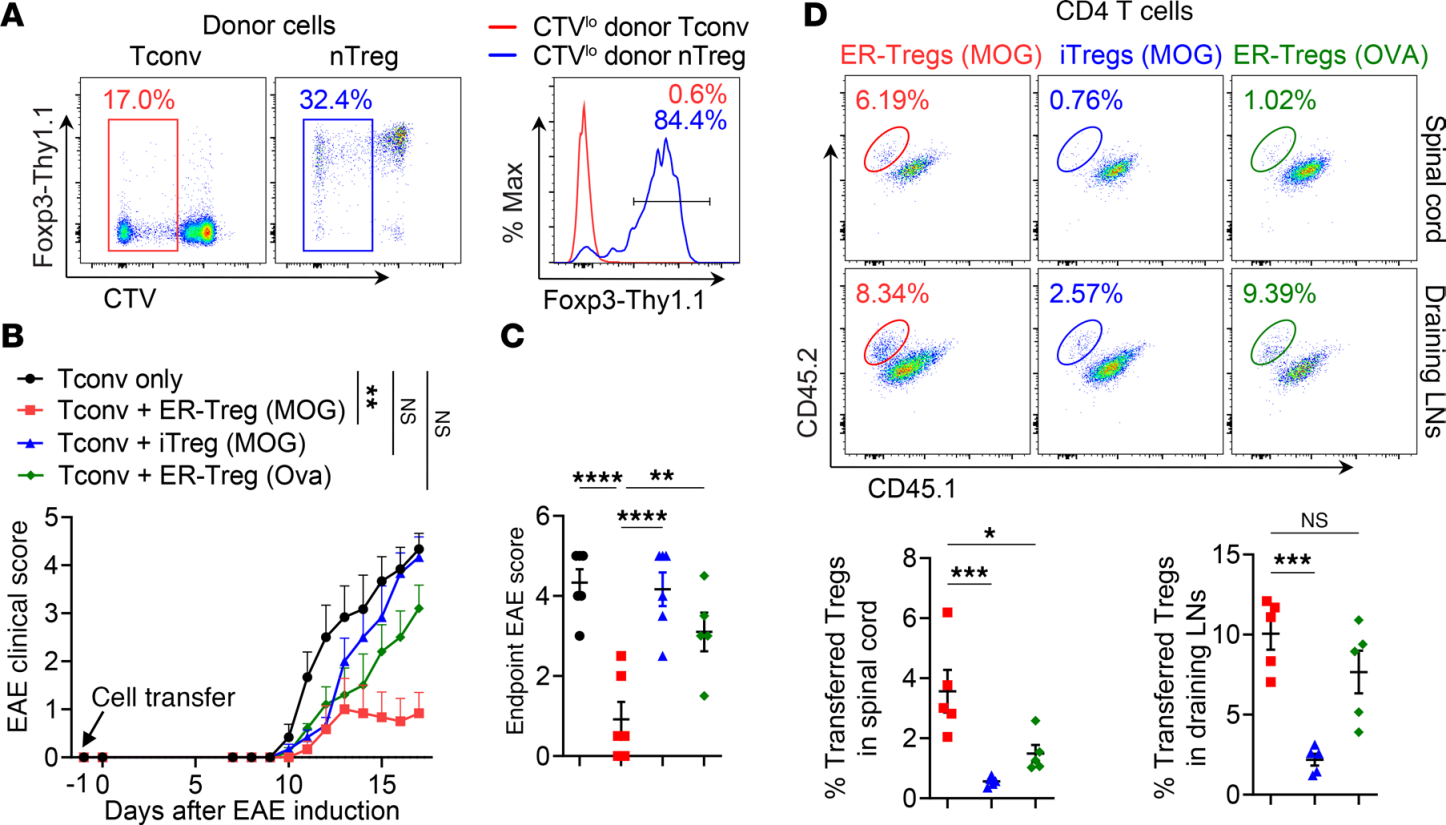
Inheritance of autoantigen specificity contributes to superior suppressive function of ER-Tregs as compared with induced Tregs. (**A**) Flow cytometry of Foxp3 expression in CTV^lo^CD4^+^ Tconv and nTregs 8 days after they were isolated from CD45.2^+^*Foxp3^Thy1.1^* mice, labeled with CTV, and adoptively transferred into CD45.1^+^*Foxp3^Thy1.1^* mice, which were subsequently immunized with MOG/CFA 1 day after adoptive transfer. (**B**–**D**) EAE was induced in *Rag1^–/–^* mice via MOG/CFA immunization 1 day after adoptive transfer of MOG/CFA-primed CD4^+^ Tconv cells with or without cotransfer of congenically distinct ER-Tregs reprogrammed from MOG/CFA- or OVA/CFA-primed CD4^+^ Teff cells, or cotransfer of induced Tregs (iTregs) generated with in vitro differentiation of Tn cells isolated from MOG/CFA-primed mice. Flow cytometry analyses were conducted at 17 dpi. *n* = 6 per group. (**B**) EAE disease curve. (**C**) EAE scores at 17 dpi. (**D**) Flow cytometry analysis of the frequencies of adoptively transferred ER-Tregs or iTregs (CD45.1^–^CD45.2^+^) in the spinal cord and draining LNs. Data are shown as mean ± SEM. *P < 0.05, **P < 0.01, ***P < 0.001, ****P < 0.0001, unpaired 2-tailed *t* test in **B**, 1-way ANOVA and Holm-Šídák test in **C** and **D**.

Antigen specificity is critical for Treg suppressor function, suggesting that the inheritance of parental Teff autoantigen specificity may enhance ER-Treg activity. To investigate this possibility, we examined whether the proinflammatory environment during autoimmune inflammation hinders the de novo differentiation of MOG-specific Tregs in EAE. We adoptively transferred CellTrace Violet–labeled (CTV-labeled) Foxp3-Thy1.1^−^ conventional CD4^+^ T (Tconv) cells or Foxp3-Thy1.1^+^ nTregs from unimmunized donor mice into CD45 congenically distinct mice, followed by immunization with MOG/CFA. Fewer than 1% of CTV^lo^ donor Tconv cells expressed Foxp3-Thy1.1, whereas the majority of CTV^lo^ donor nTregs maintained Foxp3 expression (Figure 4A), indicating that de novo differentiation of Tconv cells into Tregs does not occur in EAE. Together, these findings suggest that the proinflammatory environment in EAE drives MOG-specific CD4^+^ naive T cells to differentiate into Teff cells rather than Tregs.

To assess whether inherited myelin autoantigen specificity contributes to ER-Treg suppressor function, we performed adoptive transfers into *Rag1^–/–^* mice. Specifically, we transferred MOG/CFA-primed CD4^+^ Tconv cells alone or cotransferred them with CD45 congenically distinct ER-Tregs reprogrammed from CD4^+^ Teff cells primed in vivo with either MOG/CFA or Ovalbumin (OVA) peptide emulsified in CFA. We also included iTregs differentiated from Tn cells from MOG/CFA-immunized mice. Following recipient immunization with MOG/CFA and PT administration, mice receiving only Tconv cells developed severe EAE (Figure 4, B and C). In contrast, cotransfer of ER-Tregs derived from MOG/CFA-primed CD4^+^ Teff cells substantially mitigated EAE, whereas cotransfer of ER-Tregs derived from OVA/CFA-primed CD4^+^ Teff cells or iTregs did not. Notably, the suppressive efficacy of the transferred Tregs correlated positively with their frequencies in the spinal cord (Figure 4D). These findings suggest that inherited myelin autoantigen specificity contributes to the ability of ER-Tregs to curtail EAE development, at least in part by enhancing their ability to accrue in the inflamed tissue. Conversely, the diminished suppressive capacity of autologous iTregs may reflect diminished autoantigen specificity, as inflammatory conditions preferentially drive the differentiation of autoreactive Tn cells into Teff cells.

### ER-Tregs suppress EAE in an autoantigen-specific manner without inhibiting immune response against a nonmyelin foreign antigen.

**Figure 5:**
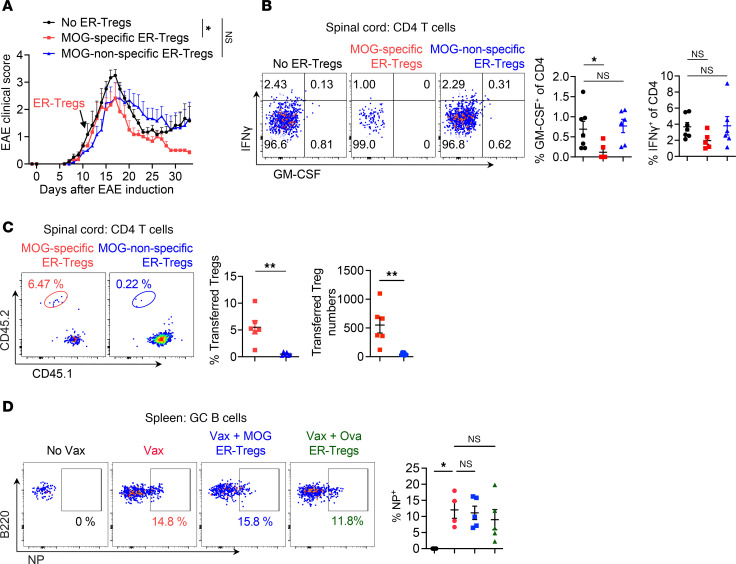
ER-Tregs confer autoantigen-specific suppression of EAE without compromising vaccine-elicited immune responses against a foreign antigen. (**A**–**C**) EAE was induced via MOG/CFA immunization in CD45.1^+^ mice with or without adoptive transfer of CD45.2^+^*Foxp3^Thy1.1^* MOG-specific or MOG-nonspecific ER-Tregs, administered at 11 dpi. Flow cytometry analyses were conducted at 33 dpi. *n* = 6 per group. (**A**) EAE disease curve. (**B**) Flow cytometry of IFN-γ and GM-CSF expression in CD4^+^ T cells in the spinal cord. (**C**) Flow cytometry analysis of the frequencies and total numbers of adoptively transferred ER-Tregs (CD45.1^–^CD45.2^+^) in the spinal cord. (**D**) Flow cytometry analysis of the frequencies of NP-specific (NP-PE^+^) germinal center B cells in the spleens of mice 11 dpi with NP-OVA/Alum with or without adoptive transfer of MOG-specific or OVA-specific ER-Tregs administered 1 day prior to immunization. Data are shown as mean ± SEM. *P < 0.05, **P < 0.01, 1-way ANOVA and Holm-Šídák test in **A**, **B**, and **D**, and unpaired 2-tailed *t* test in **C**.

Initial adoptive transfer experiments revealed that both iTregs and OVA-specific ER-Tregs transiently delayed EAE progression at early stages (Figure 4B), suggesting the possibility of antigen-nonspecific immunosuppressive effects. To clarify this, we developed a refined reprogramming protocol leveraging MOG-induced CTV dilution to isolate MOG-specific from MOG-nonspecific ER-Treg populations. Transfer of MOG-specific ER-Tregs, but not their nonspecific counterparts, significantly attenuated EAE severity and reduced spinal cord infiltration by GM-CSF^+^ CD4^+^ Teff cells (Figure 5, A and B). Moreover, MOG-specific ER-Tregs exhibited enhanced tissue fitness, as evidenced by substantially higher frequencies and total numbers in the spinal cord (Figure 5C), despite comparable splenic engraftment (Supplemental Figure 3A). They also demonstrated superior lineage stability, with elevated frequencies of Foxp3-Thy1.1^+^ cells and higher Thy1.1 MFI, as well as enrichment for c-MAF^+^RORγt^+^ subsets (Supplemental Figure 3, B and C), a phenotype linked to enhanced Th17 suppression (31–34). These findings align with previous studies showing that TCR activation enhances Foxp3 expression, functional specialization, and tissue homing in Tregs (67, 68), and they underscore the necessity of myelin antigen specificity for ER-Tregs to durably suppress CNS inflammation.

To evaluate antigen-nonspecific immunosuppression, we transferred ER-Tregs generated from either CFA/MOG- or CFA/OVA-primed Teff cells into mice immunized with nitrophenol-conjugated OVA (NP-OVA) in Alum. Neither MOG-specific nor OVA-specific ER-Tregs significantly inhibited NP-specific germinal center B cell responses (Figure 5D), although OVA-specific ER-Tregs exhibited a modest, nonsignificant trend toward suppression. These results suggest that ER-Treg–mediated suppression is tightly restricted to their cognate antigen and relies on the inflammatory context.

### ER-Tregs selectively inherit parental Teff gene expression.

**Figure 6:**
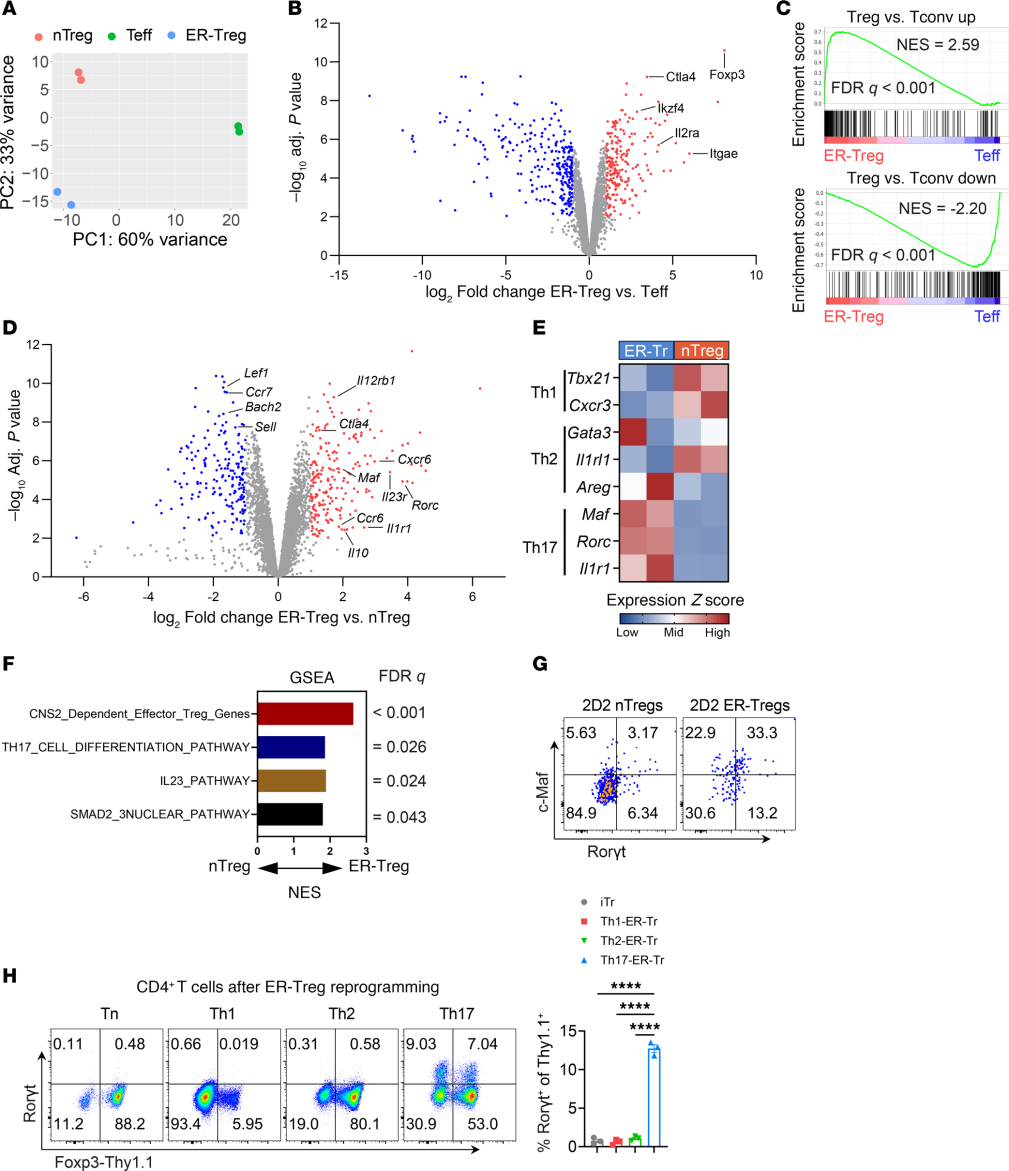
ER-Tregs exhibit elevated expression of select parental Teff genes. (**A**–**F**) RNA-Seq analysis of the transcriptomes of ER-Tregs, nTregs, and CD4^+^ Teff cells. CD45.1^+^*Foxp3^Thy1.1^* ER-Tregs were adoptively transferred into CD45.2^+^*Foxp3^Thy1.1^* mice 1 day prior to MOG/CFA immunization. The transcriptomes of transferred ER-Tregs and host nTreg and CD4^+^ Teff cells were determined by bulk RNA-Seq at 7 dpi. (**A**) Principal component analysis of ER-Treg, nTreg, and CD4^+^ Teff transcriptomes. (**B**) Volcano plot showing the differential expression of genes between ER-Tregs and Teff cells. (**C**) Gene set enrichment analysis (GSEA) of the expression of Treg-specific genes in ER-Tregs and nTregs. (**D**) Volcano plot showing the differential expression of genes between ER-Tregs and nTregs. (**E**) Normalized gene expression levels for selected lists of T helper genes in ER-Tregs and nTregs. (**F**) GSEA of the expression of indicated gene sets in ER-Tregs and nTregs. (**G**) Flow cytometry of c-Maf and RORγt expression in MOG/CFA-primed 2D2 nTregs and ER-Tregs following in vitro activation in the presence of IL-2 for 3 days. (**H**) Flow cytometry of RORγt and Foxp3-Thy1.1 expression in CD4^+^ Tn cells and in vitro differentiated T helper cells following their 1° stimulation under the ER-Treg reprogramming condition. Data are shown as mean ± SEM. ****P < 0.0001, 1-way ANOVA and Holm-Šídák test in **H**.

To determine whether parental Teff gene expression confers a distinct transcriptional profile to ER-Tregs, we performed bulk RNA-Seq on ER-Tregs, nTregs, and CD4^+^ Teff cells — all isolated from MOG/CFA-immunized mice to ensure uniform in vivo exposure. Principal component analysis (PCA) revealed that the ER-Treg transcriptome more closely resembles that of nTregs than CD4^+^ Teff cells (Figure 6A). Moreover, ER-Tregs expressed significantly higher levels of core Treg identity genes, including *Foxp3*, *Itgae*, *Il2ra*, *Ctla4*, and *Ikzf4* (22), compared with CD4^+^ Teff cells (Figure 6B). Gene set enrichment analysis (GSEA) further demonstrated that genes typically upregulated (or downregulated) in Tregs relative to CD4^+^ Tconv cells are similarly upregulated (or downregulated) in ER-Tregs relative to CD4^+^ Teff cells (Figure 6C), confirming the successful establishment of a Treg gene expression program in ER-Tregs.

Comparative transcriptomic analysis of ER-Tregs and nTregs reveals that ER-Tregs exhibit reduced expression of genes linked to T cell quiescence, such as *Lef1*, *Ccr7*, *Bach2*, and *Sell*, and increased expression of Treg effector genes, including *Ctla4* and *Il10* (69), as well as Th17-associated genes like *Rorc*, *Maf*, *Il23r*, *Ccr6*, and *Il1r1* (70, 71) (Figure 6D). Moreover, ER-Tregs express high levels of Th17 markers but not those typical of Th1 or Th2 cells (Figure 6E). GSEA further indicates that ER-Tregs upregulate genes involved in effector Treg function, Th17 differentiation, and the IL-23 pathway (Figure 6F), supporting the notion of enhanced Th17 polarization in ER-Tregs derived from MOG/CFA-primed Teff cells. Additionally, the observed amplification of SMAD2/3 signaling in ER-Tregs suggests that TGF-β in the reprogramming cocktail significantly contributes to their unique gene expression program.

ER-Tregs bearing the MOG specific 2D2 TCR express higher levels of c-Maf and RORγt compared with nTregs with the same 2D2 TCR (72) (Figure 6G), indicating enhanced Th17 polarization in myelin autoantigen-specific ER-Tregs. To assess the contribution of parental Teff gene expression to this Th17 polarization, we compared RORγt levels in ER-Tregs reprogrammed from in vitro differentiated Th1, Th2, and Th17 cells, as well as in iTregs derived from naive T cells. ER-Tregs reprogrammed from Th17 cells exhibited significantly higher RORγt expression than those reprogrammed from Th1 cells, Th2 cells, or iTregs (Figure 6H), suggesting that the inheritance of parental Th17 characteristics drives the elevated expression of selective Th17 genes in ER-Tregs.

### Elevated expression of Th17 genes contributes to ER-Treg fitness and function in EAE.

**Figure 7:**
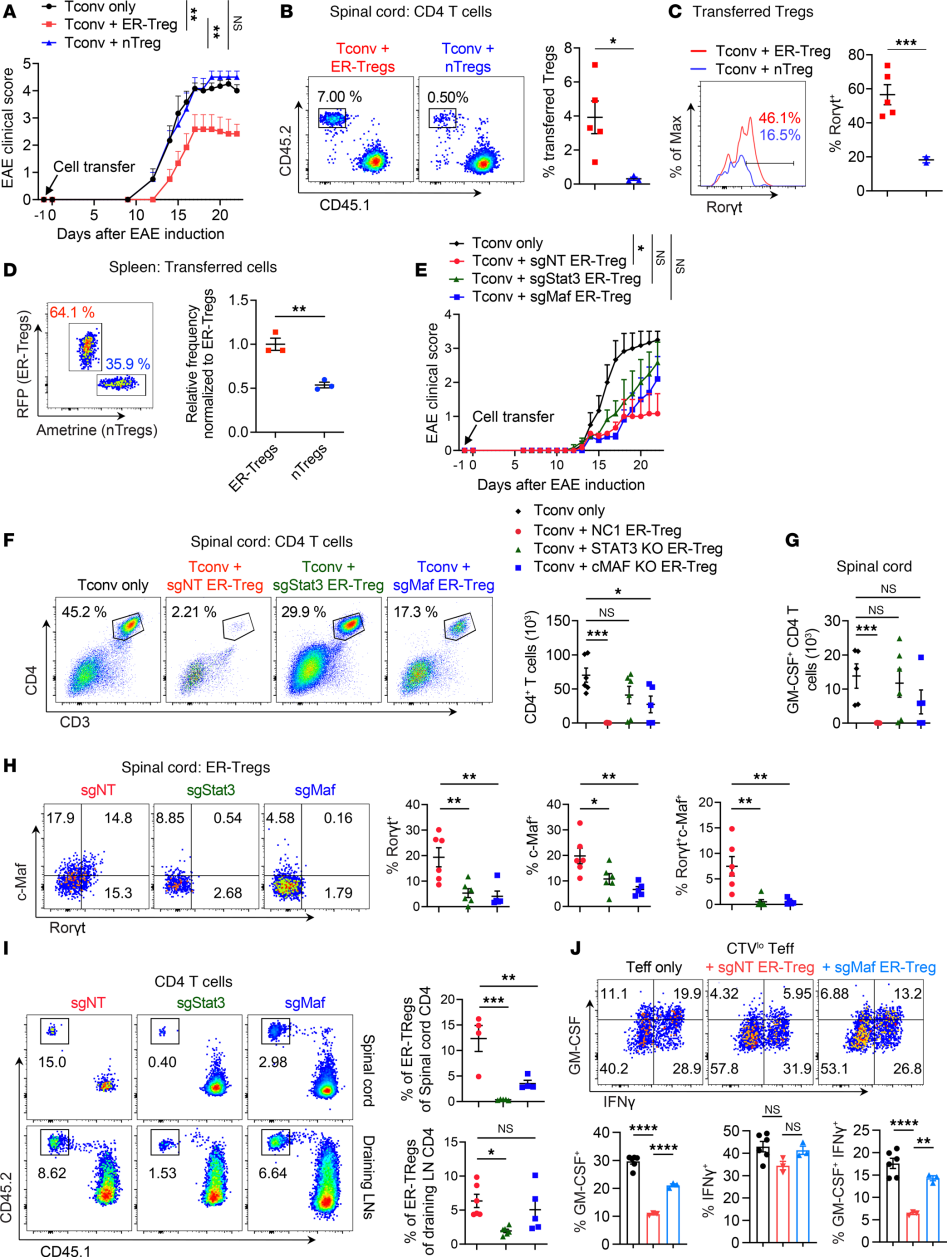
Elevated expression of specific Teff genes contributes to ER-Treg fitness and suppressive function in EAE. (**A**–**C**) EAE was induced in *Rag1^–/–^* mice 1 day after transfer of MOG/CFA-primed CD45.1^+^ CD4^+^ Tconv cells with or without cotransfer of CD45.2^+^ ER-Tregs or CD45.2^+^ nTregs isolated from MOG/CFA-primed mice and cultured in the presence of IL-2 for 3 days. Flow cytometry analyses were conducted at 22 dpi. *n* = 6 per group. (**A**) EAE disease curve. (**B**) Flow cytometry of transferred Treg frequencies. (**C**) Flow cytometry of RORγt expression in transferred Tregs. (**D**) Flow cytometry of in vivo competitive fitness between fluorescent reporter transduced 2D2 ER-Tregs and nTregs, cotransferred at a 1:1 ratio 1 day prior to CFA/MOG immunization and analyzed 5 dpi. (**E**–**I**) EAE was induced in *Rag1^–/–^* mice 1 day after transfer of MOG/CFA-primed CD45.1^+^ CD4^+^ Tconv cells with or without cotransfer of CD45.2^+^*Foxp3^Thy1.1^R26^Cas9^* ER-Tregs transduced with sgRNA-RV targeting *Stat3* (sgStat3), *Maf* (sgMaf), or a nontargeting sgRNA-RV (sgNT). Flow cytometry analyses were conducted at 22 dpi. *n* = 6–7 per group. (**E**) EAE disease curve. (**F**) Flow cytometry of the frequencies (left) and numbers (right) of CD4^+^ T cells in the spinal cord. (**G**) Numbers of GM-CSF^+^ CD4^+^ T cells in the spinal cord. (**H**) Flow cytometry of c-Maf and RORγt expression in draining LN ER-Tregs._._ (**I**) Flow cytometry of the frequencies and numbers of transferred ER-Tregs in CD4^+^ T cells in the spinal cord (upper) and draining LNs (lower). (**J**) Flow cytometry of IFN-γ and GM-CSF expression in CTV^lo^CD4^+^ Teff cells cocultured for 3 days with APCs and MOG in the presence or absence of sgMaf-RV or sgNT-RV transduced *Foxp3^Thy1.1^R26^Cas9^* ER-Tregs. Data are shown as mean ± SEM. *P < 0.05, **P < 0.01, ***P < 0.001, ****P < 0.0001, 1-way ANOVA and Holm-Šídák test in **A**, **E**, and **F**–**J** and unpaired 2-tailed *t* test in **B**–**D**.

Adoptive transfer of a limited number of ER-Tregs (2 × 10^6^ per mouse) into lymphoreplete mice harboring endogenous nTregs significantly ameliorated EAE (Figure 2), demonstrating the superior suppressive capacity of ER-Tregs over endogenous nTregs. To directly compare their therapeutic efficacy, we transferred MOG/CFA-primed CD4^+^ Tconv cells into *Rag1^–/–^* mice either alone or alongside ER-Tregs (derived from MOG/CFA-primed Teff cells) or nTregs (isolated from MOG/CFA-immunized mice). Recipients were immunized with MOG/CFA and treated with PT to induce EAE. ER-Treg cotransfer, but not nTreg cotransfer, effectively suppressed disease progression (Figure 7A). Moreover, ER-Tregs exhibited significantly greater accumulation and elevated RORγt expression in the spinal cord compared with nTregs (Figure 7, B and C), suggesting that retained Th17-associated transcriptional programming enhances their tissue fitness.

To directly evaluate the role of Th17-related gene expression in ER-Treg fitness, we performed a competitive fitness assay using ER-Tregs and nTregs from 2D2 MOG-specific mice. Both cell types were cultured under identical reprogramming conditions, transduced with distinct fluorescent reporters, and cotransferred at a 1:1 ratio into MOG/CFA-immunized mice. ER-Tregs outcompeted nTregs in vivo (Figure 7D), confirming that Th17-associated gene signatures enhance their survival and expansion. Collectively, these findings indicate that the inherited Th17-related transcriptional program underpins the enhanced fitness and suppressive efficacy of ER-Tregs relative to nTregs in EAE.

To determine whether heightened Th17 polarization underpins ER-Treg functionality in EAE, we used CRISPR/Cas9 to delete the Th17-associated transcription factors STAT3 or c-Maf in ER-Tregs and assessed their suppressive capacity. Cotransfer of control sgNT-transduced *Foxp3^Thy1.1^R26^Cas9^* ER-Tregs robustly attenuated EAE progression, whereas ER-Tregs lacking STAT3 (sgStat3) or c-Maf (sgMaf) failed to suppress disease (Figure 7E). Genetic ablation of *Stat3* or *Maf* impaired the ability of ER-Tregs to reduce spinal cord infiltration by total CD4^+^ T cells and GM-CSF^+^CD4^+^ Teff cells (Figure 7, F and G), which was accompanied by diminished expression of RORγt and c-Maf in ER-Tregs (Figure 7H). Although deletion of *Stat3* or *Maf* reduced the frequency of ER-Tregs in the spinal cord (Figure 7I), their absolute numbers remained unchanged or even elevated, respectively (Supplemental Figure 4A), likely due to increased CD4^+^ T cell accumulation in mice receiving KO ER-Tregs (Figure 7E). Notably, Foxp3 expression levels remained consistent across all groups (Supplemental Figure 4B), ruling out gross instability. These findings establish STAT3 and c-Maf as critical drivers of ER-Treg fitness and function, enabling their suppression of neuroinflammation via Th17-associated transcriptional programs.

To determine whether heightened Th17 polarization enhances ER-Treg suppressor function on a per-cell basis, we performed in vitro suppression assays comparing *Maf*-deficient ER-Tregs to control ER-Tregs transduced with sgNT. Genetic ablation of *Maf* significantly impaired the ability of ER-Tregs to suppress GM-CSF^+^CD4^+^ Teff cell responses (Figure 7J), indicating that elevated *Maf* expression is essential for their per-cell suppressive potency. Together, these results demonstrate that Th17-skewed transcriptional program in ER-Tregs is critical for their superior tissue fitness and functional efficacy in EAE compared with nTregs.

## Discussion

Endogenous Tregs acquire their identity through tolerogenic signals that imprint Treg-specific transcriptional and epigenetic programs onto naive precursors. However, whether such programs can be stably established in committed CD4^+^ Teff cells, which retain inflammatory epigenetic memory, remains unknown. To address this, we developed an epigenetic reprogramming strategy to activate core Treg transcriptional circuitry in Teff cells, creating a model system to probe Treg plasticity and therapeutic potential.

*Foxp3* induction in Teff cells is achieved through the synergistic actions of TGF-β, RA, and VC, each contributing distinct mechanistic pathways. TGF-β drives Foxp3 expression via Smad3 binding to the CNS1 enhancer of the *Foxp3* locus (73). RA amplifies this process by enhancing TGF-β/Smad3 signaling while concurrently suppressing inflammatory pathways: it downregulates IL-6 and IL-23 receptor expression, neutralizes cytokine-mediated inhibition of *Foxp3* expression, and reduces proinflammatory cytokine secretion by Teff cells (74–77). VC stabilizes *Foxp3* expression by promoting TET enzyme–dependent DNA demethylation at TSDRs, including the *Foxp3* locus itself (38, 39, 78). Stable *Foxp3* induction further requires restimulation, which sustains transcriptional activation of *Foxp3* and facilitates demethylation of the CNS2 enhancer (49), establishing a self-reinforcing loop to stabilize Treg identity. Notably, our reprogramming approach recapitulates the epigenetic remodeling observed in endogenous Tregs, inducing robust DNA demethylation at TSDRs of key Treg identity genes such as *Ctla4*, *Il2ra*, and *Ikzf4*. Crucially, this demethylation occurs independently of Foxp3 (16), indicating that TGF-β, RA, and VC cooperatively remodel the epigenome of Teff cells to activate Treg transcriptional programs through both Foxp3-dependent and Foxp3-independent mechanisms. These findings highlight the ability of tolerogenic signals to override inflammatory epigenetic memory in Teff cells, enabling their conversion into functionally stable Tregs.

Previous studies have established that ectopic Foxp3 expression in conventional CD4^+^ T cells — composed of both naive and effector T cells — can confer suppressive activity (17–19).

However, the use of conventional T cells complicates interpretation of these results. Naive T cells lack preexistent inflammatory phenotypes and, thus, are more amenable to acquiring a suppressive phenotype via Foxp3 overexpression. These Foxp3 overexpressing naive T cells could mask the resistance of Teff cells, which do have a preexistent inflammatory phenotype and epigenome, toward acquiring a suppressive phenotype. Whether Foxp3 expression alone suffices to induce authentic Treg phenotypes and suppressive function in Teff cells has, thus, remained unresolved. Our findings reveal that both stable Foxp3 induction and demethylation of Foxp3-independent Treg identity genes are indispensable for establishing functional Treg programs in Teff cells. Foxp3 critically suppresses IL-17A expression in reprogrammed cells, mirroring its role in pTregs (60), while also enhancing IL-10 production, potentially amplifying their suppressive capacity. Notably, forced Foxp3 expression in Teff cells, without concurrent epigenetic reprogramming, failed to confer suppressor function, underscoring the necessity of Foxp3-independent epigenetic remodeling at Treg identity loci. This aligns with prior work demonstrating that Treg-specific DNA demethylation, unattainable through Foxp3 overexpression alone, is essential for Treg functionality (16). Consistent with this, Foxp3-expressing Teff cells exhibited diminished expression of CD25, CTLA-4, and Helios — proteins encoded by genes harboring TSDRs (16) — suggesting that these epigenetic modifications regulate key aspects of Treg gene expression. Collectively, our work highlights a dual requirement for Foxp3 and TSDR-driven epigenetic activation to override Teff cell transcriptional programs and enforce stable Treg identity. Future studies are warranted to dissect the precise contributions of individual TSDRs to Treg transcriptional programs and their therapeutic potential in reprogramming Teff cells to treat autoimmune inflammation.

While MOG-nonspecific ER-Tregs alone failed to ameliorate established EAE, bystander suppression mechanisms — potentially mediated by nonspecific ER-Tregs in the presence of MOG-specific counterparts — may contribute to disease control (79, 80). Notably, molecular mimicry between myelin antigens and foreign antigens (e.g., Epstein-Barr virus, gut microbiota) has emerged as a key driver of multiple sclerosis (MS)/EAE pathogenesis (81–83). Although MOG-specific ER-Tregs did not impair OVA-specific vaccine responses, they may suppress immunity against microbial antigens sharing epitopes with autoantigens. These findings highlight the need to rigorously assess the long-term effects of autoantigen-specific ER-Treg therapy on immune homeostasis and infection resilience.

In contrast to ER-Tregs, adoptive transfer of MOG/CFA-primed nTregs failed to suppress EAE induced by cotransferred MOG/CFA-primed Tconv cells in *Rag1^–/–^* mice. While prior studies reported EAE mitigation using nTregs isolated from naive mice or recovery-phase mice (84–86), these protocols transferred nTregs into naive hosts at disease induction — a context lacking preexisting Teff cell differentiation and inflammation. By contrast, our cotransfer model in *Rag1^–/–^* mice recapitulates the challenge of suppressing primed Teff cells in an inflammatory milieu. Furthermore, MOG/CFA immunization destabilizes MOG-specific nTreg lineage commitment (7), likely impairing their suppressive capacity upon isolation and transfer. This instability, coupled with intrinsic limitations in effector Treg differentiation, may explain nTreg inefficacy in our model and prior studies (87). Future studies reprogramming destabilized nTregs could clarify their functional potential in EAE suppression.

We observed heightened expression of RORγt and c-Maf in ER-Tregs compared with nTregs, even when both cell types expressed the same MOG-specific 2D2 TCR. This suggests that the upregulation of RORγt expression in endogenous nTregs, which has been recently shown to be associated with the ability of nTregs to suppress Th17 inflammation in EAE (33), is hindered by the preexisting gene expression and/or epigenetic landscape in nTregs. This phenomenon may serve as a regulatory mechanism preventing endogenous Tregs from impeding beneficial antipathogen immune responses, while potentially contributing to the development of pathogenic autoimmune inflammation under specific conditions. Further investigations into the molecular mechanisms underlying the constrained or delayed Th17 polarization of nTregs in EAE and its effect on disease progression are warranted and may yield insights into the dysfunction of endogenous Tregs in Th17 cell–driven autoimmune diseases.

ER-Tregs reprogrammed from MOG/CFA-primed CD4^+^ Teff cells exhibit a unique transcriptional profile, characterized by elevated expression of Th17-associated genes (*Rorc*, *Maf*) compared with nTregs. CRISPR-mediated deletion of *Maf* or *Stat3* — key Th17 transcription factors — significantly impaired ER-Treg survival and suppressive function in EAE, highlighting the critical role of Th17-like polarization in their therapeutic efficacy. While prior studies suggest that Tregs expressing lineage-specific transcription factors (e.g., Th1, Th17) display enhanced suppression of corresponding Teff subsets (23–30), the mechanistic basis remains unclear. Our data suggest that improved cellular fitness, mediated by transcription factor–driven adaptation to inflammatory niches, may underpin this phenomenon.

The heightened Th17 signature in ER-Tregs may arise from epigenetic inheritance of parental Th17 cell programs or de novo activation during reprogramming. ER-Tregs also displayed amplified SMAD2/3 signaling, indicative of enhanced TGF-β activity, which likely drives *Maf* and *Rorc* expression — both established TGF-β targets in Tregs (31, 34, 88, 89). Given the inclusion of TGF-β in the reprogramming cocktail, these findings underscore how lineage-specific epigenetic memory synergizes with extrinsic signals to shape ER-Treg functionality. Future studies should delineate how epigenetic landscapes of distinct Th subsets influence ER-Treg differentiation and function, particularly in inflammatory contexts.

Our study highlights the superior in vivo fitness of ER-Tregs over other Treg subsets, driven by intrinsic properties like their polarization state. CRISPR-mediated ablation of c-Maf impaired ER-Treg suppression of GM-CSF^+^ Teff cells in vitro, suggesting that Th17 polarization enhances their per-cell suppressive potency. While APC interactions were not directly assessed, bulk RNA-Seq revealed elevated *Ctla4* expression in ER-Tregs (Figure 6D). Given the established role of CTLA-4 in downregulating CD80/CD86 on APCs via transendocytosis (90–93), this upregulation likely contributes to their enhanced suppressive function. Beyond EAE, Th17-derived ER-Tregs — combining stable Foxp3 expression, robust tissue persistence, and potent suppression — hold promise for other Th17-driven diseases such as inflammatory bowel disease. More broadly, our epigenetic reprogramming strategy could be applied to other helper subsets; for example, converting autoreactive Th1 cells into stable Tregs may offer a targeted approach to mitigate pancreatic β cell autoimmunity in type 1 diabetes.

Our study establishes that coordinated demethylation of *Foxp3* and Foxp3-independent Treg identity genes enables the conversion of committed CD4^+^ Teff cells into functional Tregs with bona fide transcriptional and suppressive programs. The resulting ER-Tregs outperform endogenous nTregs, iTregs, and Foxp3-overexpressing Teff cells in suppressing established autoimmune inflammation, underscoring their therapeutic potential. This enhanced efficacy arises from dual mechanisms: (a) inherited autoantigen specificity, which promotes antigen-specific and tissue-localized suppression, and (b) retention of parental Teff transcriptional programs, which bolsters fitness in inflammatory niches. These insights advance our understanding of Treg epigenetic regulation while offering a blueprint for engineering antigen-specific Treg therapies with tailored functionality to treat autoimmune diseases.

## Methods

### Sex as a biological variable.

Though women are more susceptible to developing MS than men by a ratio of approximately 3:1, men who develop MS exhibit greater cognitive impairment and more rapid disability progression than women. Our study examined both male and female mice, and similar findings are reported for both sexes.

### Mice.

Animals were housed at the TUSM animal facility under specific pathogen–free conditions according to institutional guidelines. All mouse strains used were on the C57BL/6 genetic background. *CD45.1* (no. 002014), *Rosa26^Cas9-eGFP^* (no. 026179), *2D2* (no. 006912), and *Rag1^–/–^* (no. 002216) mice were purchased from The Jackson Laboratory. *Foxp3^Thy1.1^* mice were a gift from Y. Zheng. The above mouse strains were bred in-house at TUSM to produce the *Foxp3^Thy1.1^ Rosa26^Cas9^*, *Foxp3^Thy1.1^ CD45.1^+/+^*, and *2D2^+/–^ Foxp3^Thy1.1^ CD45.1^+/–^* mouse strains. Male and female mice used were at least 6 weeks old and had no prior exposure to drugs or experimentation.

### Antibodies and reagents.

Flow cytometry antibodies anti-CD3 (2C11), anti-CD4 (RM4-5), anti-Thy1.1 (HIS51), anti-CD44 (IM7), anti-CD62L (MEL-14), anti-CD45.1 (A20), anti-CD45.2 (clone 104), anti–IFN-γ (XMG1.2), anti-GMCSF (MP1-22E9), anti-B220 (RA3-6B2), anti-GL7 (GL7), anti-CD138 (clone 281-2), anti-CXCR5 (L138D7), and anti-NGFR (ME20.4) were purchased from BioLegend. Anti–IL-17 (eBio17B7), anti-Foxp3 (FJK-16s), anti-RORγt (B2D), and anti–c-MAF (sym0F1) were purchased from eBioscience. Anti-CD95 (Jo2) was purchased from BD Biosciences. Neutralizing antibodies toward IFN-γ (XMG1.2), IL-4 (11B11), and IL-12 (C17.8) were purchased from BioXcell. Human IL-2 and IL-7 were purchased from PeproTech. Mouse TGF-β, IL-6, IL-23, and IL-1b were purchased from R&D Systems. RA was purchased from Sigma-Aldrich. VC was purchased from Thermo Fisher Scientific. CD3/CD28 Dynabeads were purchased from Thermo Fisher Scientific. Incomplete Fruend’s Adjuvant was purchased from Thermo Fisher Scientific. MOG (amino acids 35–55) was purchased from GeneMed Synthesis. Heat-killed *M. tuberculosis* strain H37 Ra was purchased from BD Biosciences.

### ER-Treg generation.

CD4^+^CD44^hi^ Teff were sort purified from donor mice that were immunized with CFA/MOG 7 days prior. Sorted cells were rested in T cell growth medium (RPMI 1640 [Corning] supplemented with 2 mM GlutaMAX [Gibco], 10 mM HEPES [Gibco], 100 U/mL penicillin/streptomycin [Corning], 1 mM sodium pyruvate [Corning], 5% fetal calf serum [Gemini Bioproducts]) for 4 days in the presence of 2 ng/mL IL-7 and 10 μg/mL neutralizing antibodies against IFN-γ, IL-4, and IL-12. Cells were then stimulated with CD3/CD28 Dynabeads for 4 days in T cell growth medium supplemented with 1,000 U/mL IL-2, 5 ng/mL TGF-β, 100 μg/mL VC, 10 nM RA, and 10 μg/mL cytokine neutralizing antibodies (primary reprogramming cocktail). CD4^+^Foxp3-Thy1.1^+^ cells were sort purified and restimulated with Dynabeads in T cell growth medium supplemented with 1,000 U/mL IL-2, 2 ng/mL TGF-β, 10 μg/mL VC, and 10 μg/mL cytokine neutralizing antibodies (secondary reprogramming cocktail). For experiments using transduced ER-Tregs, CD4^+^Foxp3-Thy1.1^+^Reporter^+^ cells were sort purified and restimulated.

### Retroviral vectors.

MG2A and MG2N were generated by modifying MSCV-P2GM-FF (plasmid no. 19750, Addgene). Mouse *Foxp3*, *Maf*, *Rorc*, and *Stat3* single guide RNAs (sgRNA) were cloned into BbsI-digested MG2N or MG2A. MIGR-mFoxp3 was a gift from D. Littman (plasmid no. 24067, Addgene). The guide sequences are nontargeting (NT) (5′-GCACTACCAGAGCTAACTCA-3′), *Foxp3* (5′-GTTCCTGGGTGTACCCGAGCG-3′), *Maf* (5′-GCCCGCAGCAGCTCAACCCGG-3′), *Rorc* (5′-GTCATCTGGGATCCACTACG-3′), and *Stat3* (5′-GAGATTATGAAACACCAACG-3′).

### Production of retrovirus.

Retrovirus was produced by transfecting HEK293T cells (ATCC) 2 days prior to transduction using the pCL-Eco (Addgene plasmid no. 12371) packaging vector and Fugene HD transfection reagent (Promega). Medium was replaced with half the transfection volume 1 day before transduction.

### Retroviral transduction.

Rested CD4^+^ Teff cells were stimulated for 1 day with CD3/CD28 Dynabeads in the presence of the primary reprogramming cocktail. Cells were then transduced by spin infection with viral supernatant supplemented with the primary reprogramming cocktail and 4 μg/mL polybrene (Sigma-Aldrich). Spin infection was performed in a Sorvall Legend X1R centrifuge for 90 minutes at 1,113*g* and 37°C.

### Bisulfite sequencing.

Genomic DNA was isolated using the GeneJet Genomic DNA Purification Kit (Thermo Fisher Scientific) and then bisulfute converted using EpiTect Bisufite Conversion Kit (Qiagen). Converted DNA was amplified with Q5U polymerase (New England Biolabs) and gel purified after agarose gel electrophoresis. Purified PCR product was cloned into pJET1.2 (Thermo Fisher Scientific) for Sanger sequencing. The bisulfite amplification primers are CNS2 forward (5′-TGGGTTTTTTTGGTATTTAAGAAAG-3′), CNS2 reverse (5′-AACCAACCAACTTCCTACACTATCTAT-3′), CTLA4 forward (5′-TGGTGTTGGTTAGTAGTTATGGTGT-3′), CTLA4 reverse (5′-AAATTCCACCTTACAAAAATACAATC-3′), IL2ra forward (5′-TTTTAGAGTTAGAAGATAGAAGGTATGGAA-3′), IL2ra reverse (5′-TCCCAATACTTAACAAAACCACATAT-3′), Ikzf4 forward (5′-AGGATGGTTTTTATTGAAGGTGAT-3′), Ikzf4 reverse (5′-ATACACACCAAACAAACACTACACC-3′).

### EAE induction.

EAE was induced by subcutaneous injection of 50 μL of an emulsion containing 50 μg MOG_35–55_ and 250 μg *M*. *tuberculosis* strain H37 Ra in Incomplete Fruend’s Adjuvant into each hind flank. Mice also received an intraperitoneal injection of 200 ng PT in 200 μL PBS (Corning) on days 0 and 2 after immunization. Clinical signs of EAE were assessed by the following criteria: 0, no signs of disease; 1, loss of tail tone; 2, hind limb paresis; 3, hind limb paralysis; 4, tetraplegia; 5, moribund or dead. Mice with a score greater than 4 were euthanized and carried with a score of 5 for the duration of the experiment.

### Cell transfer.

For all preventive EAE experiments in *Rag1^–/–^* mice, cells were transferred i.v. 1 day prior to disease initiation. Mice received 50,000 Tregs and 100,000 CD4^+^ Tconv from CD45 congenically distinct *Foxp3^Thy1.1^* donor mice. CD4^+^ Tconv were procured from mice that were immunized with CFA/MOG 7 days prior to transfer using the mouse CD4^+^ T Cell Isolation Kit (Miltenyi Biotec) followed by Treg depletion using anti-Thy1.1 PE (HIS51) and anti-PE nanobeads (BioLegend). For preventive EAE experiments in lymphoreplete mice, 2 × 10^6^ Tregs from CD45 congenically distinct *Foxp3^Thy1.1^* donor mice were transferred i.v. 1 day prior to disease initiation. For therapeutic EAE experiments in lymphoreplete mice, 2 × 10^6^ polyclonal or 0.5 × 10^6^ MOG-specific CD45 congenically distinct Tregs were transferred i.v. at first evidence of disease (tail paralysis, typically day 11 after disease initiation). For in vivo fitness experiments in lymphoreplete mice, 0.5 × 10^6^ 2D2 TCR transgenic CD45 congenically distinct Tregs were transferred i.v. 1 day prior to immunization with MOG/CFA.

### Cell isolation.

For lymph node and spleen, tissues were mechanically dissociated using the back of a syringe plunger and filtered through a 70 μm nylon mesh. Spinal cords were harvested by perfusing mice with PBS and collecting the tissue. Spinal cords were then cut into small pieces and enzymatically digested with 1 mg/mL collagenase D and 0.1 mg/mL DNase (Sigma-Aldrich) in T cell growth medium for 30 minutes at 37°C with shaking at 1,500 rpm. Tissue digests were filtered through a 40 μm nylon mesh and remaining tissue was mechanically dissociated with the back of a syringe plunger. Dissociated spinal cord cells were then placed into a 30%-37%-70% isotonic Percoll (Cytiva) gradient and centrifuged for 30 minutes at room temperature at 800*g* to enrich the infiltrating mononuclear cells.

### Flow cytometry.

For surface staining, cells were stained in FACS buffer (PBS, 0.5% BSA, 1mM EDTA) for 15 minutes at 4°C, washed, and analyzed. For intracellular cytokine staining, cells were incubated at 37°C in the presence of 50 ng/mL PMA (LC laboratories) and 500 ng/mL ionomycin (LC laboratories) for 1 hour. GolgiStop (BD Biosciences) was added, and the cells were incubated at 37°C for an additional 3 hours. Stimulated cells were surface stained, fixed, and permeabilized with Foxp3/Transcription Factor Staining Kit (Tonbo Biosciences) according to manufacturer instructions, and they were stained for cytokines in permeabilization buffer. Washed cells were stored in FACS buffer until analysis.

### In vitro suppression assays.

CD45 congenically distinct CD4^+^CD44^hi^ Teff cells were sorted from donor mice immunized 7–10 days prior and labeled with CTV (Invitrogen). Labeled cells were cocultured with T cell–depleted APCs and Tregs in the presence of 10 μg/mL MOG_35–55_. Dilution of CTV and cytokine expression were measured 4 days later.

### RNA-Seq.

CD45 congenically distinct ER-Tregs were transferred to lymphoreplete mice, and the mice were immunized with CFA/MOG. After 7 days, ER-Tregs, nTregs, and CD4^+^ Teff cells were sorted from the draining lymph nodes directly into TRIzol (Qiagen). RNA was isolated using phenol-chloroform extraction. Uniquely indexed libraries were pooled in equimolar ratios and sequenced on a single Illumina NextSeq500 run with single-end 75 bp reads by the Tufts University Genomics Core Facilities.

### Transcriptome analysis.

Sequence reads were aligned with the mm39 reference genome assembly and gene counts were quantified with FeatureCounts. Differential expression analysis was performed with DESeq2. GSEA were performed with GSEAPreranked, in which genes ranked according to their fold changes were compared with the following MSigDB signature collections: GSE7852_Treg_VS_Tconv_DN gene set, GSE7852_Treg_VS_Tconv_UP gene set, WP_TH17_CELL_DIFFERENTIATION_PATHWAY, PID_IL23_PATHWAY, PID_SMAD2_3NUCLEAR_PATHWAY, as well as a CNS2_Dependent_Effector_Treg gene set generated from GSE57272.

### Statistics.

Except for RNA-Seq analysis, statistical significance was determined using GraphPad Prism 10.0 (GraphPad Software). For comparisons of a single variable between 2 groups, significance was determined using 2-tailed unpaired *t* tests. For comparisons of multiple groups where variance did not significantly differ across groups, 1- or 2-way ANOVA with Šídák’s (for comparisons between preselected pairs) multiple-comparison corrections was used. EAE disease scores were analyzed with 1-way ANOVA of AUC of clinical scores. *P* values below 0.05 were considered statistically significant and are shown by the exact number or by asterisks in the figures.

### Study approval.

All studies were performed under protocol no. B2022-85 and approved by TUSM IACUC.

### Data availability.

All data are available in the main text or the supplemental materials. Individual data point values can be found in the Supporting Data Values. RNA-Seq data can be accessed using GEO accession no. GSE303685. All materials used or generated in this study are available to researchers following appropriate standard material transfer agreements.

## Author contributions

Conceptualization was contributed by XL and TRC. Methodology was contributed by XL and TRC. Formal analysis was contributed by TRC. Investigation was contributed by TRC, JJC, HIM, MN, JLL, JHK, ANP, and XL. Writing was contributed by XL and TRC. Visualization was contributed by TRC. Supervision was contributed by XL. Project administration was contributed by XL. Funding acquisition was contributed by XL.

## Supplementary Material

Supplemental data

Supporting data values

## Version 1 07/31/2025

In-Press Preview

## Version 2 09/23/2025

Electronic publication
